# Cancer associated fibroblasts-derived SULF1 promotes gastric cancer metastasis and CDDP resistance through the TGFBR3-mediated TGF-β signaling pathway

**DOI:** 10.1038/s41420-024-01882-y

**Published:** 2024-03-04

**Authors:** Xingchao Fang, Damin Chen, Xinyu Yang, Xiaogang Cao, Quan Cheng, Kanghui Liu, Peng Xu, Yanjuan Wang, Jiafeng Xu, Siguo Zhao, Zhengyuan Yan

**Affiliations:** 1 Department of General Surgery, Nanjing Lishui People’s Hospital, Nanjing, Jiangsu China; 2 Department of General Surgery, the First Affiliated Hospital of Nanjing Medical University, Nanjing, Jiangsu Province China; 3 Department of Gastroenterology, The First Affiliated Hospital of Nanjing Medical University, Nanjing, Jiangsu Province China; 4 Department of Clinical Medicine, The First School of Clinical Medicine, Anhui Medical University, Hefei, Anhui China

**Keywords:** Cell invasion, Gastric cancer

## Abstract

SULF1 has been implicated in a number of malignancies. The function of SULF1 in gastric cancer is disputed. The objective of this study was to examine the role and underlying molecular mechanisms of SULF1 in the context of gastric cancer. We found that the expression of SULF1 was increased in gastric cancer, especially in cancer-associated fibroblasts. The overexpression of SULF1 was found to be significantly correlated with unfavorable prognosis among individuals diagnosed with gastric cancer. Functionally, cancer-associated fibroblasts-derived SULF1 served as a oncogenic molecule which facilitated gastric cancer cells metastasis and CDDP resistance. Mechanistically, SULF1 regulated the communication between gastric cancer cells and cancer-associated fibroblasts in tumor microenvironment as a signaling molecule. Cancer-associated fibroblasts-secreted SULF1 interfered with the interaction between TGF-β1 and TGFBR3 by combining with TGFBR3 on gastric cancer cell membrane, subsequently activated TGF-β signaling pathway. In conclusion, our findings have presented novel approaches for potential treatment and prognosis prediction in individuals diagnosed with gastric cancer through the targeting of the CAFs-SULF1-TGFBR3-TGF-β1 signaling axis.

## Introduction

Gastric cancer (GC) is a highly prevalent gastrointestinal malignancy with significant mortality rates worldwide, particularly in Eastern Asia [1]. The utilization of radical gastrectomy in conjunction with chemotherapy treatments is a prevalent approach for patients with advanced GC [2]. Chemotherapy regimens based on cisplatin (CDDP), doxorubicin, docetaxel, and fluorouracil (CDDP-based) are commonly suggested therapeutic approaches for individuals diagnosed with advanced GC [3]. The majority of individuals diagnosed with gastric cancer experienced mortality as a result of tumor recurrence and metastasis [4]. The survival rate of patients with advanced GC is further reduced due to the development of chemotherapy resistance [5]. Additional research is needed to have a comprehensive understanding of the fundamental mechanisms and molecular foundations that contribute to the advancement of gastric cancer and the development of resistance to chemotherapy.

The tumor microenvironment (TME) is a critical factor in the advancement of tumors and the development of resistance to treatment [6, 7]. It comprises various components, including stromal cells, immune cells, cytokines, the extracellular matrix, and the blood vascular network [7, 8]. Cancer-associated fibroblasts (CAFs) constitute the principal component of the TME, contributing significantly to the extracellular matrix and intercellular signaling molecules [9–11]. CAFs engage in intercellular communication by modulating the extracellular matrix and releasing various signaling molecules such as cytokines, chemokines, and exosomes [12, 13]. CAFs are commonly characterized by their elevated levels of fibroblast activation protein (FAP) and alpha-smooth muscle actin (α-SMA) expression [10]. Multiple investigations have documented the participation of CAFs in the progression of GC, including tumor growth, metastasis, and therapy resistance [14–16].

Human sulfatase 1 (SULF1) is an extracellular sulfatase characterized by the function of desulfation in heparan sulfate proteoglycans (HSPGs) on cell membrane and extracelluar matrix [17]. HSPGs, act as coreceptors consisted of heparan sulfate chains and glucosamine disaccharide units, bind receptors and signal molecules in order to assist them proper localization and interaction [18]. SULF1 desulfates HSPGs and then decreases the interaction between HSPGs with signal molecules, such as Wnts, TGF-β1 and FGFs [19–21]. The function of SULF1 in tumors was disputable, especially in GC. Several studies reported that SULF1 inhibited the proliferation of hepatocellular carcinoma, reduced the invasiveness in the head and neck squamous cell carcinoma and suppressed the chondrosarcoma growth [22–24]. Some researches demonstrated that SULF1 increased the ability of migration and invasion in hepatocellular carcinoma [21]. SULF1 was found to inhibit the proliferation and migration of GC cells, while another study reported that SULF1 predicted poor prognosis in the population with GC [25, 26]. The real role and underlying mechanisms of SULF1 in GC development need to be further clarified.

In the current investigation, a novel role of SULF1 in the intercellular communication between GC cells and CAFs was discovered. Based on the findings of our study, it was observed that the expression of SULF1 was significantly elevated in GC, particularly in the CAFs present within the microenvironment of GC. The overexpression of SULF1 was found to be correlated with unfavorable prognosis and negative clinicopathological features. The activation of the transforming growth factor-beta (TGF-β) signaling pathway in GC cells was facilitated by the interaction between SULF1, originating from CAFs, and TGFBR3 located on the cell membrane. This interaction leaded to the initiation of the epithelial-mesenchymal transition (EMT) process, metastasis, and resistance to cisplatin (CDDP) in GC cells. SULF1 played a role in mediating the communication between CAFs and GC cells, functioning as a signaling protein that is secreted by CAFs. Given its role in promoting development in GC, SULF1 exhibited potential as a viable molecular target. Inhibiting SULF1 could be a valuable strategy to impede the progression of GC and enhance the effectiveness of chemotherapy treatments.

## Results

### SULF1 was overexpressed in gastric cancer-associated fibroblasts

**Fig. 1:**
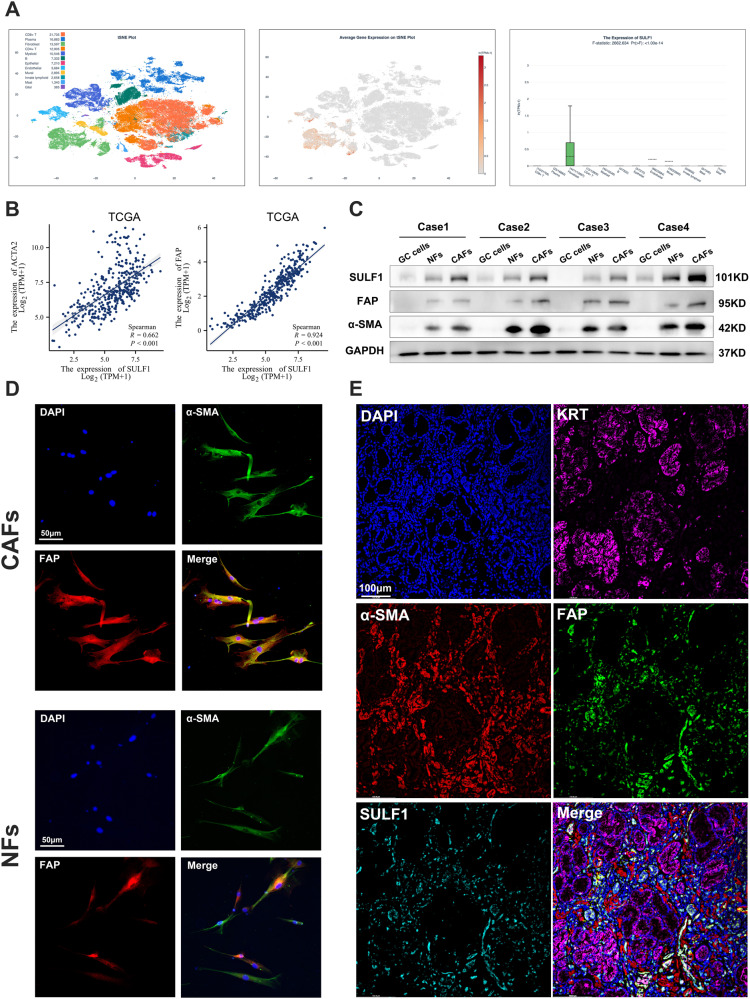
SULF1 was predominately expressed in gastric cancer-associated fibroblasts. **A** SULF1 expression analysis using single-cell RNA sequencing dataset GSE206785 from OmniBrowser database. **B** correlation analysis between SULF1 and CAFs markers, α-SMA and FAP, using GC RNA sequencing data from TCGA dataset. **C** Western blotting results to detected the expression of SULF1, α-SMA and FAP in indicated primary cells extracted from GC and paired normal tissues. **D** Immunofluorescence to detected the expression of α-SMA and FAP in NFs and CAFs. **E** Multiple tissue immunofluorescence stained SULF1 with KRT, α-SMA and FAP to detected the spatial localization of SULF1 in human GC tissues.

The role of SULF1 in tumors has been controversial. As for GC, SULF1 has been reported to be associated with poor prognosis and lymph node metastasis [25]. Hai-Meng Zhou et al. demonstrated that SULF1 inhibited GC cells proliferation and invasion [26]. Based on single-cell RNA sequencing dataset, GSE206785, we found that SULF1 was predominantly expressed in the fibroblasts of GC tissues (Fig. 1A). SULF1 exhibited significant positive correlations in mRNA expression with ACTA2 (α-SMA) and FAP, the CAFs markers, using TCGA dataset (Fig. 1B). CAFs are the predominant cell type in the tumor microenvironment and have a significant impact on a variety of malignant biological behaviors of tumor cells [9, 14]. These data suggested that SULF1 may expressed in CAFs and thus influence the development of GC. We extracted primary NFs, CAFs and GC cells from human gastric cancer and paracancerous tissues to verify the expression of SULF1. The CAFs exhibited higher expression of α-SMA and FAP than NFs as shown in immunofluorescence and western blotting assays (Fig. 1C, D). Western blotting results showed that GC cells expressed lowest SULF1, and the expression of SULF1 was higher in CAFs than NFs (Fig. 1C). Then we stained SULF1 with α-SMA and FAP in human GC tissues. As shown in Fig. 1E, SULF1 was mainly enriched in CAFs (α-SMA^+^ FAP^+^). These data demonstrated that SULF1 was predominantly expressed in CAFs of GC.

### Elevated SULF1 expression correlated with the adverse clinicopathological characteristics and poor prognosis in GC

**Fig. 2:**
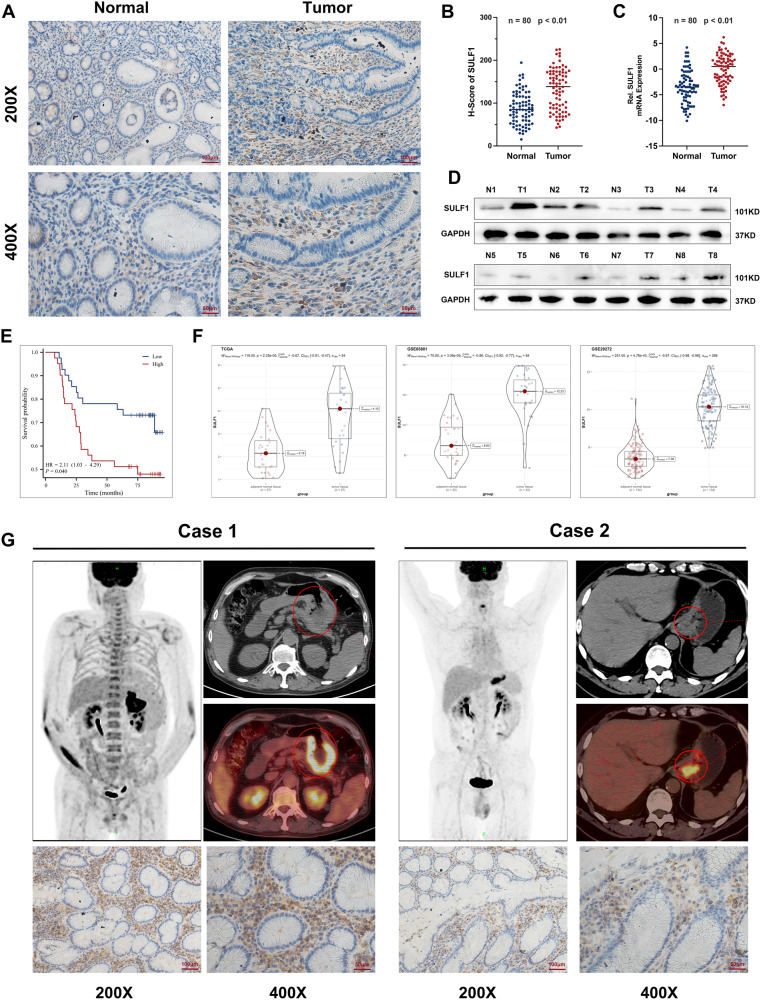
SULF1 was associated with the adverse clinicopathological characteristics and poor prognosis in GC. IHC staining (**A**) and quantitative analysis result (**B**) of SULF1 in 80 paired GC and normal tissues. **C** qRT-PCR result of SULF1 mRNA expression in 80 paired GC and normal tissues. **D** Western blotting results of SULF1 expression in 8 paired GC and normal tissues. **E** Survival analysis of SULF1 in 80 GC patients using logrank test. **F** Differential analysis of SULF1 expression in TCGA, GSE65801 and GSE29272 datasets. **G** Two representative cases of gastric cancer patients with high and low expression of SULF1 who underwent PET-CT examination.

We analyzed the expression of SULF1 in human GC and adjacent normal tissues from our center and public databases. IHC staining and qRT-PCR assays in 80 paired tissues from our center displayed that SULF1 expression was higher in GC tissues than the adjacent normal tissues (Fig. 2A–C). The same results was obtained from western blotting assays in 8 paired tumor and normal tissues (Fig. 2D). The datasets, TCGA, GSE65801 and GSE29272, further verified that SULF1 was significantly upregulated in GC (Fig. 2F). In addition, survival analysis using H-scores of SULF1 IHC staining revealed GC patients with higher SULF1 expression have shorter overall survival (Fig. 2E). It indicated that SULF1 correlated with poor prognosis in GC patients. The clinicopathological information of above 80 GC patients showed high SULF1 expression was related to large tumor size, poor tumor differentiation, lymph node metastasis and advanced tumor stage in GC patients (Table 1). The PET-CT examination images also confirmed that patients with higher SULF1 expression exhibited more advanced GC (Fig. 2G). Overall, we illustrated that SULF1 was highly expressed in gastric cancer and was associated with advance clinicopathological characteristics and poor prognosis.

**Table 1:** Relationships between the H-Score of SULF1 and clinicopathologic characteristics in 80 gastric cancer patients.

Characteristics	Group	Case	H-Score of SULF1		*p* value
			Low	High	
Age	<60	39	22 (27.5%)	17 (21.2%)	0.263
	≥60	41	18 (22.5%)	23 (28.7%)	
Gender	Male	49	23 (28.7%)	26 (32.5%)	0.491
	Female	31	17 (21.2%)	14 (17.5%)	
Tumor size	<4 cm	46	29 (36.2%)	17 (21.2%)	**0.007**
	≥4 cm	34	11 (13.8%)	23 (28.7%)	
Tumor differentiation	Well + moderate	53	31 (38.8%)	22 (27.5%)	**0.033**
	Poor	27	9 (11.2%)	18 (22.5%)	
Tumor Invasion	T1–T2	29	20 (25%)	9 (11.2%)	**0.011**
	T3–T4	51	20 (25%)	31 (38.8%)	
Lymph node metastasis	Negative	29	22 (27.5%)	7 (8.8%)	**<0.001**
	Positive	51	18 (22.5%)	33 (41.2%)	
TNM stage	I–II	33	22 (27.5%)	11 (13.8%)	**0.012**
	III–IV	47	18 (22.5%)	29 (36.2%)	

*P* < 0.05 was considered statistically significant.

### SULF1 contributed to CAFs-induced migration and invasion of GC cells

**Fig. 3:**
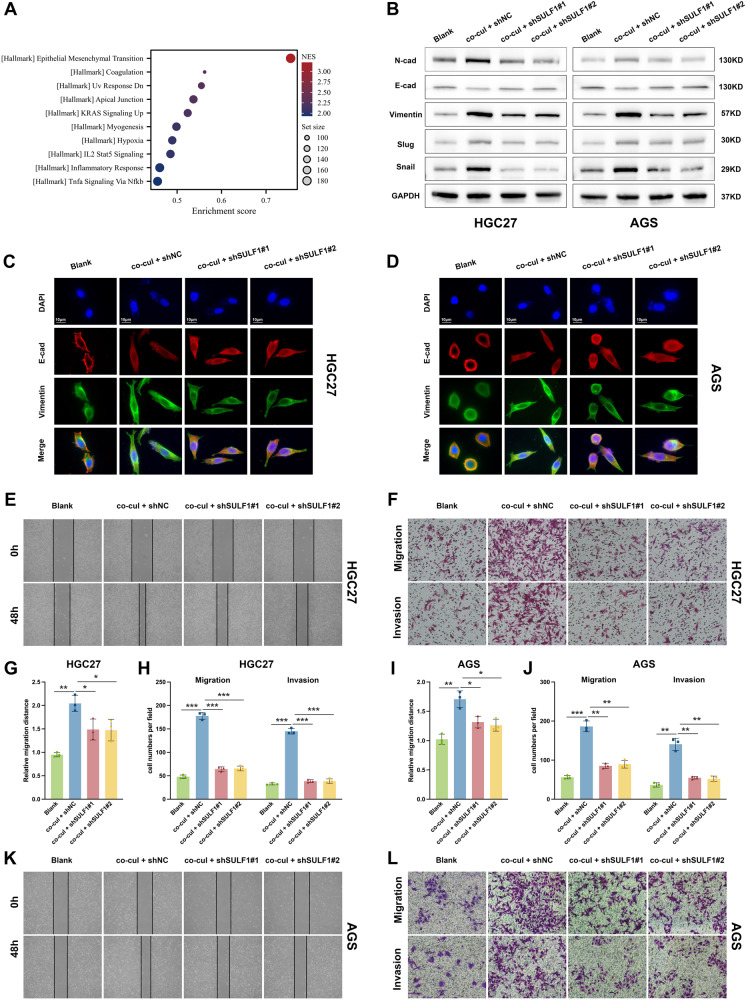
SULF1 contributed to CAFs-induced migration and invasion of GC cells. **A** The top 10 enriched signaling pathways of SULF1 using GSEA analysis. **B**. Western blotting to detected the expression of EMT markers in HGC27 and AGS cells from indicated groups. Immunofluorescence stained E-cadherin and vimentin to detected the EMT process in HGC27 (**C**) and AGS (**D**) cells from indicated groups. Representative images of wound-healing (**E**) and transwell (**F**) assays in HGC27 cells from indicated groups. Quantitative analysis results of wound-healing (**G**) and transwell (**H**) assays in HGC27 cells from indicated groups. Quantitative analysis results of wound-healing (**I**) and transwell (**J**) assays in AGS cells from indicated groups. Representative images of wound-healing (**K**) and transwell (**L**) assays in AGS cells from indicated groups.

To identify the function of cancer-associated fibroblasts-derived SULF1 in GC, we knockdowned SULF1 with lentivirus (shSULF1#1 and shSULF1#2) in human primary CAFs extracted from human GC tissues (Fig. S1A). HGC27 and AGS GC cell lines were subjected to further experiments since they barely expressed SULF1 (Fig. S1B). Then we constructed co-culture systems with CAFs and GC cells (Fig. S1C). We conducted a bulk spearman correlation analysis of SULF1 using TCGA-STAD dataset. The correlated gene list and corresponding coefficients were used for GSEA. According to the GSEA results, gene set that positively correlated with SULF1 showed highest enrichment in EMT (epithelial-mesenchymal transition) signaling pathway (Fig. 3A). Western blotting and immunofluorescence assays were preformed to testify the expression of EMT-related markers. Results showed that GC cells which co-cultured with CAFs developed EMT, while the transition was diminished when knockdown of SULF1 in CAFs (Fig. 3B–D). It is well known that EMT is a critical process in tumor metastasis [27]. Subsequently, wound-healing and transwell experiments were performed to validate the impact of SULF1 on the migratory and invasive capabilities of GC cells in an in vitro setting. The findings of the study demonstrated that CAFs significantly augmented the migratory and invasive capabilities of GC cells. The downregulation of SULF1 in CAFs resulted in a reduction in the migratory and invasive capabilities of GC cells that were produced by co-culturing with CAFs (Fig. 3E–L). In conclusion, these experiments indicated that cancer-associated fibroblast-derived SULF1 promoted the migration and invasion of GC cells.

### Cancer-associated fibroblasts-derived SULF1 activated TGF-β signaling pathway in GC cells by binding to TGFBR3

**Fig. 4:**
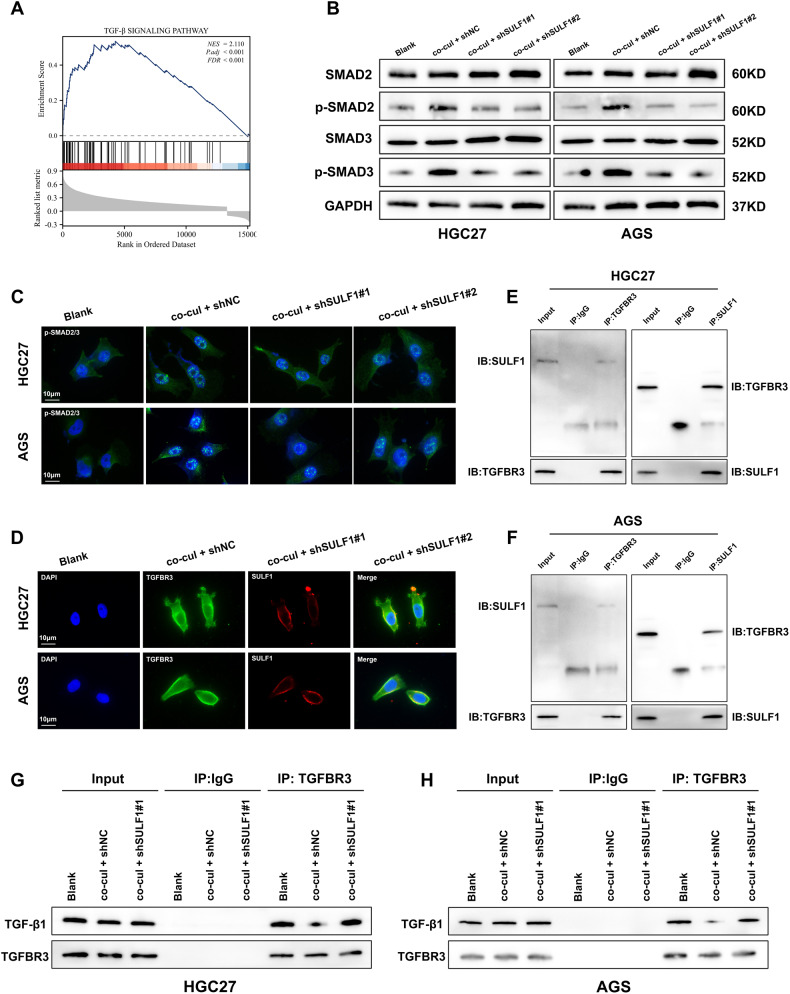
Cancer-associated fibroblasts-derived SULF1 activated TGF-β signaling pathway in GC cells by binding to TGFBR3. **A** GSEA analysis result of SULF1 in TGF-β signaling pathway. **B** Western blotting to detected the expression of SMAD2, SMAD3, p-SMAD2 and p-SMAD3 in HGC27 and AGS cells under indicated treatments. **C** Immunofluorescence to detected the nuclear localization of p-SMAD2/3 in HGC27 and AGS cells under indicated treatments. **D** Immunofluorescence to detected the co-localization of SULF1 and TGFBR3 in HGC27 and AGS cells co-cultured with CAFs. Co-IP assays to detected the interaction between SULF1 and TGFBR3 in HGC27 (**E**) and AGS (**F**) cells co-cultured with CAFs. Western blotting to detected the TGF-β1 immunoprecipitated by TGFBR3 in HGC27 (**G**) and AGS (**H**) cells under indicated treatments.

Previous study reported that SULF1 activated the TGF-β/SMAD pathway by binding to TGFBR3 and decreasing the interaction between TGF‐β1 and TGFBR3 in hepatocellular carcinoma [21]. In addition, TGF-β signaling pathway was closely related to EMT and CAFs [28, 29]. The GSEA results also verified that SULF1 was associated with the TGF-β signaling pathway (Fig. 4A). We speculated that CAFs-derived SULF1 promoted the ability of metastasis in GC cells through TGFBR3. To verify our speculation, we firstly detected the expression of SMAD2/3 and p-SMAD2/3 in GC cells under indicated treatments. As shown in western blotting and immunofluorescence, CAFs co-culture increased the expression of p-SMAD2/3 and promoted the localization of p-SMAD2/3 in nucleus, while SULF1 konckdown in CAFs diminished the function (Fig. 4B, C). Immunofluorescence in CAFs co-cultured GC cells showed that SULF1 and TGFBR3 were colocalized on the cell membrane (Fig. 4D). Co-IP assay further testify the binding of SULF1 and TGFBR3 (Fig. 4E, F). Then we performed IP assay using the first antibody against TGFBR3. Results showed that the interaction between TGF‐β1 and TGFBR3 was decreased following CAFs co-culture, while SULF1-knockdowned CAFs reversed the effects (Fig. 4G, H). Collectively, these findings demonstrated that SULF1 involved in CAFs-induced TGF-β signaling pathway activation by binding to TGFBR3.

### SULF1 contributed to CDDP resistance in GC cells

**Fig. 5:**
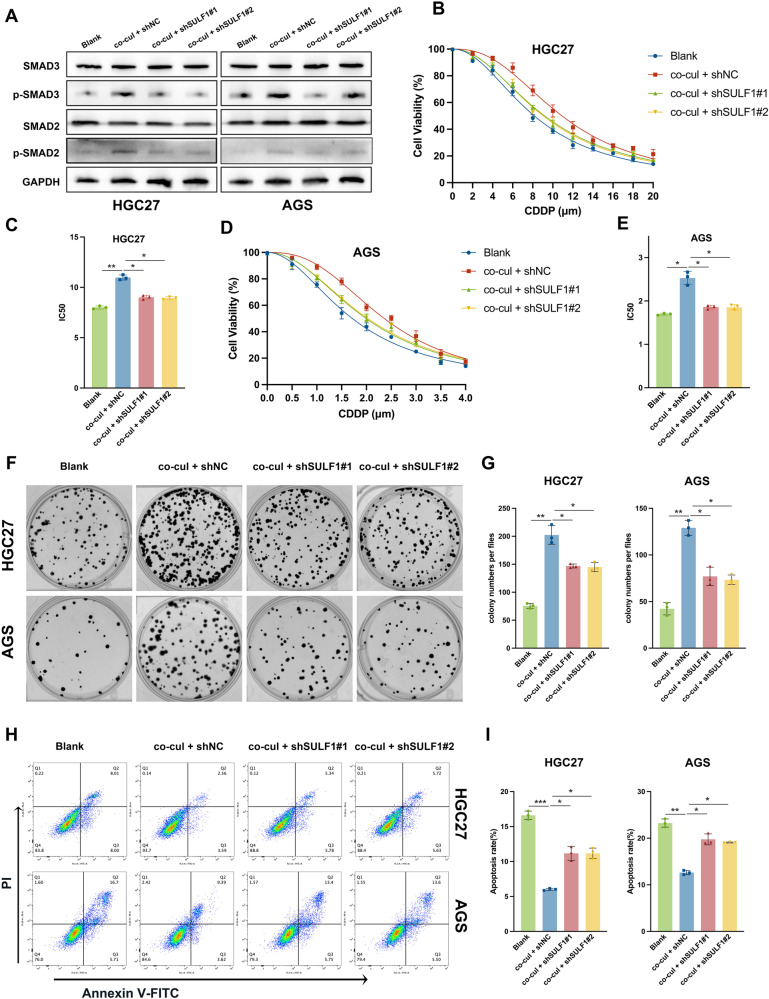
Cancer-associated fibroblasts-derived SULF1 promoted the CDDP resistance of GC cells. **A** Western blotting to detected the expression of SMAD2, SMAD3, p-SMAD2 and p-SMAD3 in CDDP treated HGC27 (8 μm; 24 h) and AGS (2 μm; 24 h) cells from indicated groups. **B** CCK8 assay to detected the cell viability of CDDP treated HGC27 cells from indicated groups. **C** Quantitative analysis results of IC50 of HGC27 cells regarding CDDP using CCK8 assay results. **D** CCK8 assay to detected the cell viability of CDDP treated AGS cells from indicated groups. **E** Quantitative analysis results of IC50 of AGS cells regarding CDDP using CCK8 assay results. Representative images (**F**) and quantitative analysis results (**G**) of colony formation assay in CDDP treated HGC27 (8 μm; 24 h) and AGS (2 μm; 24 h) cells from indicated groups. Representative images (**H**) and quantitative analysis results (**I**) of flow cytometry in CDDP treated HGC27 (8 μm; 24 h) and AGS (2 μm; 24 h) cells from indicated groups.

As reported in previous studies, CAFs and TGF-β signaling pathway are involved in chemotherapy resistance in a variety of tumors [30, 31]. Chemotherapy regimens based on CDDP are commonly indicated as therapeutic interventions for individuals diagnosed with advanced GC [3]. Considering CAFs-derived SULF1 was able to active TGF-β signaling pathway, we speculated that SULF1 could induce CDDP resistance in GC. SULF1 continued to activate the TGF-β pathway in the condition of CDDP treatment (Fig. 5A). According to the IC50 results measured by CCK8 assay, GC cell viability regarding CDDP in the co-cul + shNC group was higher than the blank group and the co-cul + shSULF1 group (Fig. 5B–E). It indicated HGC27 and AGS cells which co-cultured with CAFs exhibited stronger resistance to CDDP, while the CDDP resistance was reduced when SULF1 knockdown. Colony formation assays further demonstrated that CAFs-derived SULF1 enhanced the survival of GC cells treated with CDDP (Fig. 5F, G). We then performed flow cytometry to detect GC cells apoptosis under CDDP treatments. As shown in the Fig. 5H, I, the co-culture system protected GC cells from CDDP induced apoptosis, while SULF1 knockdown attenuated the protection of GC cells by CAFs co-culture. Taken together, these results suggested that CAFs-derived SULF1 facilitated the CDDP resistance in GC cells.

### TGF-β pathway was involved in SULF1-induced malignant phenotype of GC cells

**Fig. 6:**
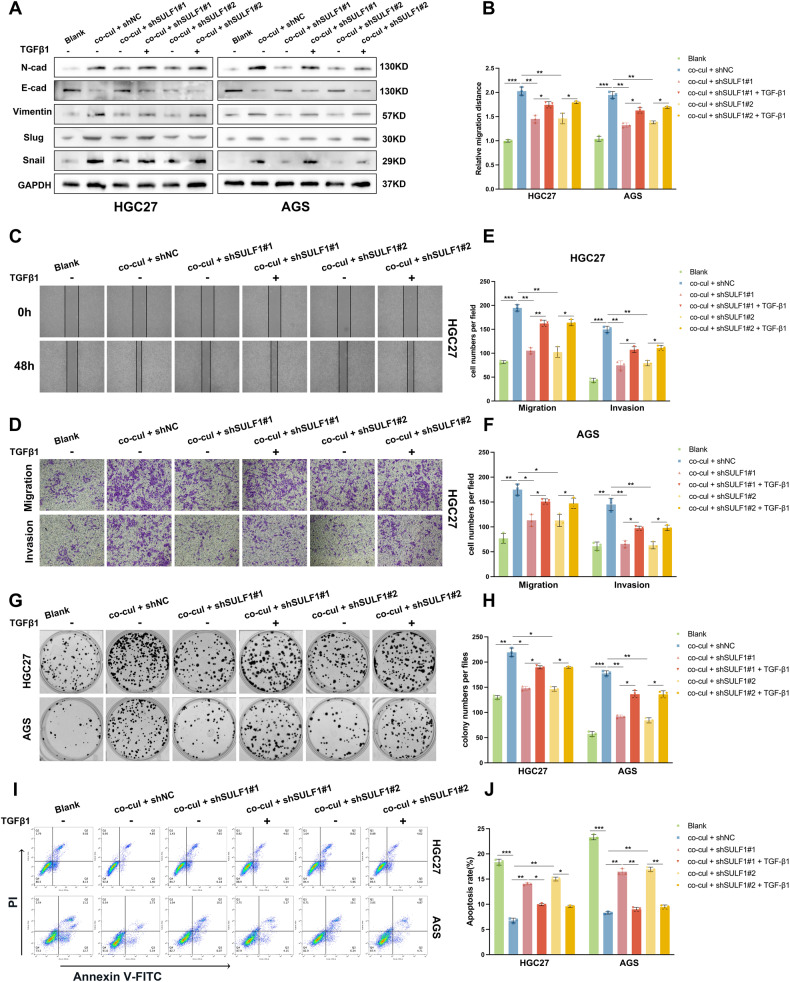
TGF-β pathway was involved in SULF1-induced malignant phenotype of GC cells. **A** Western blotting to detected the expression of EMT markers in HGC27 and AGS cells from indicated groups with or without treated with TGF-β1 (20 μg/mL) for 24 h. **B** Quantitative analysis results of wound-healing assays in HGC27 and AGS cells from indicated groups with or without treated with TGF-β1 (20 μg/mL) for 24 h. **C** Representative images of wound-healing assays in HGC27 cells from indicated groups with or without treated with TGF-β1 (20 μg/mL) for 24 h. **D** Representative images of transwell assays in HGC27 cells from indicated groups with or without treated with TGF-β1 (20 μg/mL) for 24 h. Quantitative analysis results of transwell assays in HGC27 (**E**) and AGS (**F**) cells from indicated groups with or without treated with TGF-β1 (20 μg/mL) for 24 h. Representative images (**G**) and quantitative analysis results (**H**) of colony formation assay in CDDP treated HGC27 (8 μm; 24 h) and AGS (2 μm; 24 h) cells from indicated groups with or without treated with TGF-β1 (20 μg/mL) for 24 h. Representative images (**I**) and quantitative analysis results (**J**) of flow cytometry in CDDP treated HGC27 (8 μm; 24 h) and AGS (2 μm; 24 h) cells from indicated groups with or without treated with TGF-β1 (20 μg/mL) for 24 h.

Subsequently, functional experiments were performed to validate the participation of the TGF-β pathway in the induction of a malignant phenotype of GC cells by SULF1. Western blotting results showed that EMT suppressed by knockdown of SULF1 was restored by TGF-β1 treatment (Fig. 6A). The wound-healing and transwell experiments demonstrated that the migratory and invasive capacities of GC cells treated with TGF-β1 were much higher compared to the control GC cells. (Fig. 6B–F, Fig. S2A, B). According to the CCK8 assay results, TGF-β1 treatment increased the IC50 of GC cells that co-cultured with SULF1-konckdowned CAFs (Fig. S2C, D). The colony formation and flow cytometry assay results also confirmed that TGF-β1 treatment protected GC cells that co-cultured with SULF1-konckdowned CAFs from CDDP induced apoptosis (Fig. 6G–I). Then we treated GC cells with TGF-β inhibitor and human recombinant SULF1 protein. As shown in Fig. S2E–L, hSULF1 and the corresponding control GC cells exhibited similar ability in metastasis and CDDP resistance under the treatment of SB-431542. Combined with the previous findings, we conducted that CAFs-derived SULF1 may promote metastasis and CDDP resistance of GC cells through the TGF-β pathway.

### SULF1 promoted metastasis and CDDP resistance of gastric cancer in vivo

**Fig. 7:**
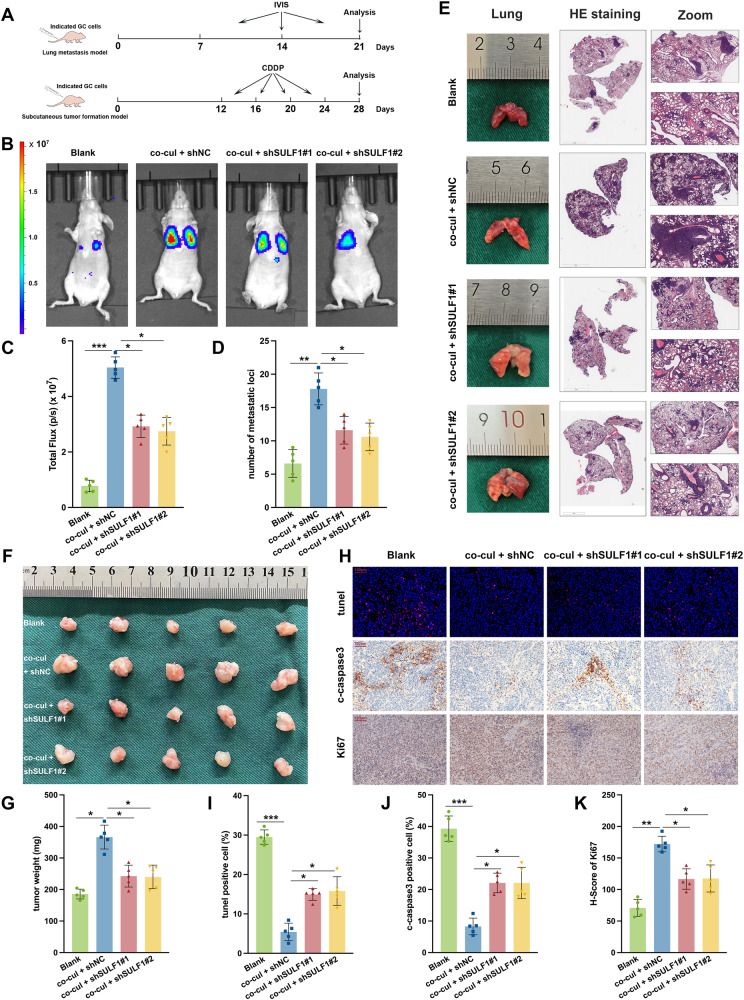
SULF1 promoted metastasis and CDDP resistance of gastric cancer in vivo. **A** The schematic diagram of animal experiments. Representative images (**B**) and quantitative analysis results (**C**) of IVIS Spectrum Imaging System in mice from indicated groups. Representative H&E staining images (**D**) and quantitative analysis results (**E**) of lung metastatic nodules from indicated groups. Representative images (**F**) and weight quantitative analysis results (**G**) of subcutaneous tumors from indicated groups. **H** Representative images of IHC stained tunel, c-caspase3 and Ki67 in subcutaneous tumors from indicated groups. Quantitative analysis results of IHC stained tunel (**I**), c-caspase3 (**J**) and Ki67 (**K**) in subcutaneous tumors from indicated groups.

In order to further validated the role of CAFs-derived SULF1 in GC, we conducted the lung metastasis model and subcutaneous tumor formation model in nude mice by inoculating HGC27 cells with indicated treatments (Fig. 7A). The IVIS Spectrum Imaging System showed that co-cultured HGC27 cells exhibited greater ability of lung metastasis, while SULF1 knockdown exhibited the negative effects (Fig. 7B, C). H&E staining also confirmed that the number of metastatic nodules was increased by CAFs co-culture but decreased by SULF1 knockdown (Fig. 7D, E). According to the subcutaneous tumor formation model results, the co-cul + shNC group displayed the biggest and heaviest tumor under CDDP treatments, while SULF1 knockdown inhibited the tumor growth (Fig. 7F, G). We then stained tunel and c-caspase3 to detect apoptosis rate in tumors from indicated groups. Results showed that CAFs co-culture protected HGC27 cells from CDDP induced apoptosis, while SULF1 knockdown weakened the efficiency (Fig. 7H–J). Ki67 staining demonstrated the same results (Fig. 7H, K). In conclusion, these results verified that SULF1 contributed to GC cell metastasis and CDDP resistance induced by CAFs.

## Discussion

As revealed by the “seed and soil” theory, the TME is necessary for multiple biological processes in tumor development and progression [32]. CAFs and their secreted extracellular matrix constitute the majority of the tumor microenvironment [10]. A variety of studies have investigated the function of CAFs in tumor growth, metastasis, recurrence, and therapeutic resistance [12, 15, 33, 34]. CAFs normally exhibited the function to communicate with tumor cells via matrix reconstruction and the secretion of cytokines, chemokines and exosomes [10]. The investigation of the underlying processes via which CAFs communicate with GC cells is of utmost importance in the context of preventing tumor progression and developing effective treatment interventions. Based on our results, we found that GC cells exhibited significantly increased ability of metastasis and CDDP resistance after cultured in CAFs supernatant. Furthermore, we identified SULF1 as a novel signaling molecule that is implicated in the intercellular communication between GC cells and CAFs. CAFs-derived SULF1 contributed to the oncogenic function of CAFs. The findings suggest that SULF1 could serve as a promising target for inhibiting the progression of gastric cancer generated by CAFs.

In our study, we identified a novel role of SULF1 in GC. Some studies reported that SULF1 was downregulated and inhibited tumor progression in several cancers. The downregulation of SULF1 promoted the malignancy of chondrosarcoma by regulating the receptor tyrosine kinase signaling [22]. In the head and neck squamous carcinoma, SULF1 acted as a negative factor in tumor cell growth [23]. In the context of GC, it has been documented that the presence of SULF1 is correlated with an unfavorable prognosis and an increased risk of lymph node metastases, while several researchers demonstrated that SULF1 inhibited GC cells proliferation and invasion [25, 26]. Based on our findings, it was revealed that GC tissues had a significant upregulation of SULF1 expression, which was found to be indicative of an unfavorable prognosis for individuals diagnosed with GC. We were the first time to identified that SULF1 was predominately enriched in gastric CAFs. In contrast to the results of some previous studies, we discovered that SULF1, secreted by CAFs, was a positive regulator for GC progression. CAFs-derived SULF1 promoted GC cells metastasis and CDDP resistance in vitro and vivo by the activation of TGF-β signaling pathway.

To summarize, this present study indicated that CAFs within gastric cancers promoted metastasis and CDDP resistance through the secretion of SULF1. The CAFs-derived SULF1 activated the TGF-β signaling pathway via the combination with TGFBR3 on GC cell surface which largely contributed to the oncogenic function of SULF1. According to our findings, the signaling axis involving CAFs-SULF1-TGFBR3-TGF-β1 has emerged as a potentially valuable target for prognosis prediction and treatment intervention in individuals with GC.

## Materials and methods

### Patients and tissue specimens

All the 80 paired gastric cancer and adjacent normal tissues were collected from Nanjing Lishui People’s Hospital during 2015–2016. These patients underwent no chemotherapy and radiotherapy. In addition, corresponding clinicopathological information including age, sex, tumor size, tumor differentiation, lymph node metastasis and tumor stage were collected. The research was approved by the Medical Ethics Committee of Nanjing Lishui People’s Hospital and the written-informed consent was acquired from all patients.

### Primary cells extraction

Primary GC cells, CAFs and NFs were extracted from GC and paired normal tissues by the outgrowth method. The fresh tissues were minced into 1 mm^3^ and cultured with the DMEM medium (Wisent, 319-020-CL) supplied with 10% fetal bovine serum (FBS) (Gibco, 10270–106) at 37 °C and 5% CO_2_. The medium was changed every 3 days. Fibroblasts and tumor cells were separated according to the principle of adherent difference. CAFs were further identified by the spindle-shaped morphology and CAF-specific markers (α-SMA^+^ and FAP^+^). The 2nd to 5th generations of CAFs were used for further studies.

### Cell culture and transfection

The human gastric mucosal epithelial cell GES-1 and GC cell lines SNU-1, HGC27, SNU-719, MKN28, MKN-45 and AGS were purchased from Cell Bank of the Chinese Academy of Sciences (Shanghai, China). They were cultured in the condition of 5% CO_2_ and 37 °C, with RPMI-1640 medium (Wisent, 350-000-CL) contained 10% FBS. Primary GC cells, CAFs and NFs were cultured in DMEM contained 10% FBS.

In order to establish stable CAFs that knockdown SULF1, shRNA (GenePharma, China) sequences against SULF1 (shSULF1#1 and shSULF1#2) were packaged in lentivirus vectors. Sequences used for RNAi were listed in supplementary Table S1.

### Co-culture system

The schematic diagram of co-culture system with CAFs and GC cells was exhibited in Fig. S1C. Briefly, 1 × 10^5^ GC cells and 1 × 10^5^ CAFs were respectively seeded into the lower and upper chambers of a six-well Transwell apparatus with 0.4 µm pore (Corning Incorporated). In some experiments, cisplatin with indicated concentrations was added to the co-culture system. Passage was conducted when cell grow to 90%. After one week of co-culture, the GC cells were collected for further studies.

### Quantitative reverse transcription-polymerase chain reaction (qRT-PCR)

The total RNA was extracted as previous study [35]. Reverse transcription was performed using HiScript III All-in-one RT SuperMix Perfect for qPCR (Vazyme, R333-01) according to the manufacturer’s protocols. RT‒qPCR was performed with AceQ qPCR SYBR Green Master Mix (Vazyme, Q141-02) by using QuantStudio 7 system (Thermo Fisher Scientific, USA). The ΔCt method was used to quantify relative mRNA expression, which was normalized to GAPDH.

Primers sequences used for qRT-PCR were listed in supplementary Table S2.

### Western blotting

The method of protein extraction and western blotting was performed as previous study [35]. The ImageJ software was used to quantify relative protein expression, which was normalized to GAPDH.

Antibodies used for western blotting were listed in supplementary Table S3.

### Co-Immunoprecipitation (Co-IP)

For Co-IP assay, cell lysates were incubated with primary antibodies at 4 °C overnight. Next day, the mixture was incubated with the Protein A + G Magnetic Beads (Beyotime, P2179) for 1 h at room temperature. After the beads were washed, the immunoprecipitated protein were resuspended in SDS loading buffer and then boiled for 5 min at 95 °C. The samples were detected by western blotting.

Antibodies used for Co-IP were listed in supplementary Table S6.

### Animal studies

All the 4-week-old female BALB/C nude mice were bought from the Animal Experimental Center of Nanjing Medical University. The animal experiments were approved by the Committee on the Ethics of Animal Experiments of Nanjing Medical University. Mice were randomly divided into indicated groups (5 mice for each group). For the lung metastasis model, 1.5 × 10^6^ HGC27 cells were injected into mice via tail vein with or without 0.5 × 10^6^ stable transfected CAFs. In vivo imaging system (IVIS) was performed every 7 days to detected the HGC27 cells metastasis. 3 weeks after injection, mice were sacrificed and the collected lung tissues were subjected to HE staining. For the subcutaneous tumor formation model, 1.5 × 10^6^ HGC27 cells were injected into the forelimb axilla of 6 weeks-old nude mice with or without 0.5 × 10^6^ stable transfected CAFs. 12 days later, mice were intraperitoneally injected with 5 mg/kg cisplatin every 4 days. Tumor volume was calculated every 4 days with the following formula: *V* = *π*/6 (Length × Width^2^) after cisplatin injection. 4 weeks after cells injection, mice were sacrificed and collected tumor tissues were weighted.

### Bioinformatics and statistics

Single-cell RNA sequencing analysis was preformed using OmniBrowser database (https://omnibrowser.abiosciences.cn/#/browser/dashboard). GSEA, survival analysis, correlation analysis and expression difference analysis of SULF1 using public datasets were performed with RStudio (1.4.1717).

All studies were conducted three times independently and the results were shown as mean ± SD. *P* < 0.05 were considered statistically significant. SPSS 22.0 and GraphPad Prism 9.0 software were used for statistical analysis. Student’s *t*-test or one-way ANOVA were used to analyze the statistically significant difference between different groups.

### Supplementary information

Supplemental Figures

Supplemental Materials

Supplemental Tables

Original Data File

## Data Availability

The authors declare that all data supporting the findings of this study are available with the article or from the corresponding author upon reasonable request.
