# Supplementary material for: Cancer associated fibroblasts-derived SULF1 promotes gastric cancer metastasis and CDDP resistance through the TGFBR3-mediated TGF-β signaling pathway
Source: Cell Death Discov. 2024 Mar 4;10:111. doi: 10.1038/s41420-024-01882-y (PMC10912303; doi:10.1038/s41420-024-01882-y)
Supplement: Supplementary file 1 — Supplemental Figures [file 41420_2024_1882_MOESM1_ESM.docx]

**Supplemental Figures**

**
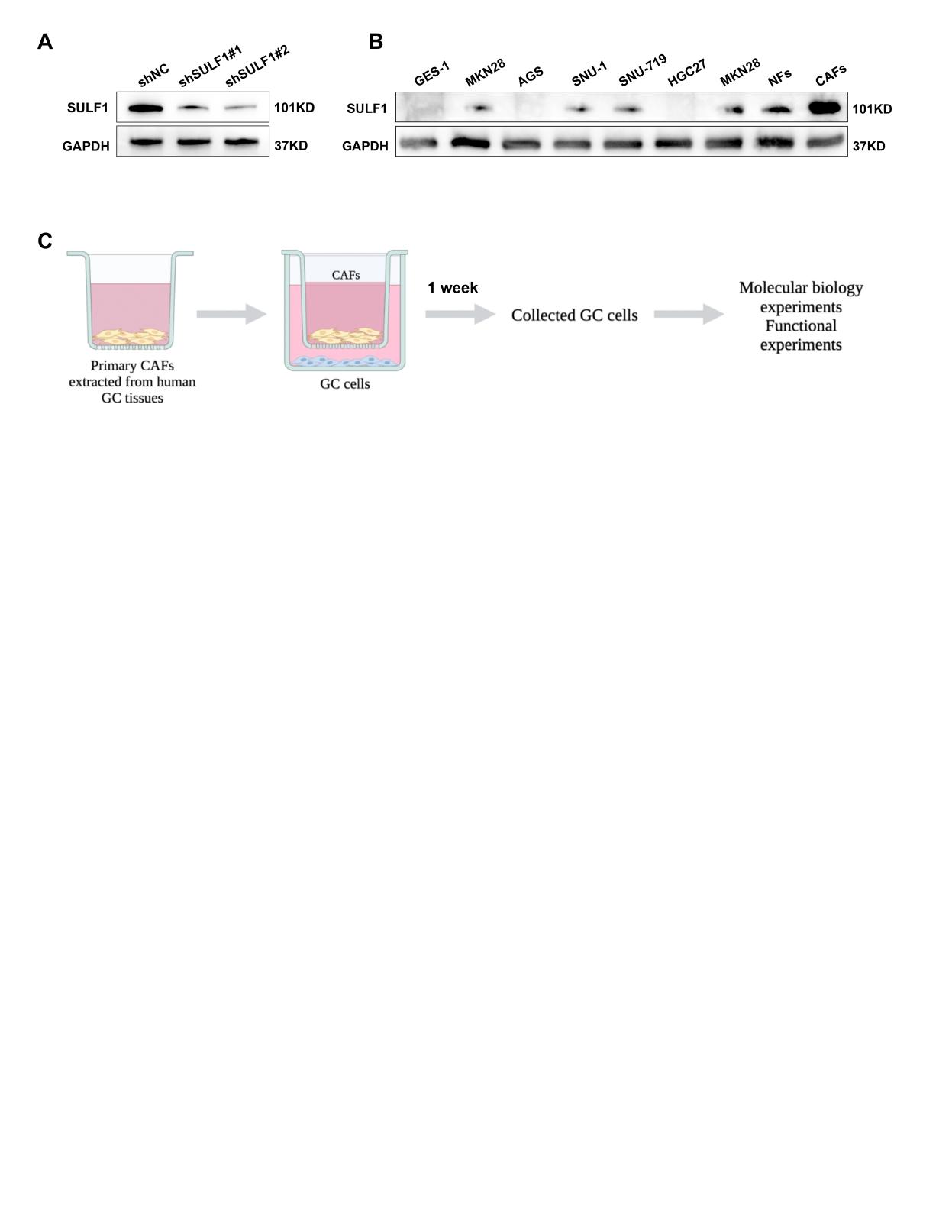
**

**Fig. S1**: Additional results of **Fig. 1**

1. Western blotting to detected the SULF1 expression in CAFs following indicated lentivirus infected. **B**. Western blotting to detected the SULF1 expression in indicated GC and normal mucosal epithelial cells. **C**. The schematic diagram of co-culture system with CAFs and GC cells.

**Fig. S2**: Additional results of **Fig. 6**

**A**. Representative images of wound-healing assays in AGS cells from indicated groups with or without treated with TGF-β1 (20 μg/mL) for 24 h. **B**. Representative images of transwell assays in AGS cells from indicated groups with or without treated with TGF-β1 (20 μg/mL) for 24 h. **C**. Quantitative analysis results of IC50 of HGC27 cells regarding CDDP from indicated groups with or without treated with TGF-β1 (20 μg/mL) for 24 h. **D**. Quantitative analysis results of IC50 of AGS cells regarding CDDP from indicated groups with or without treated with TGF-β1 (20 μg/mL) for 24 h. **E and F.** Representative images of transwell assays in HGC27 and AGS cells with indicated treatments of hSULF1 (5 μg/mL, 24h) and SB-431542 (5 μM, 24h). **G and H.** Quantitative analysis results of transwell assays in HGC27 and AGS cells with indicated treatments of hSULF1 (5 μg/mL, 24h) and SB-431542 (5 μM, 24h). **I and J.** Representative images and quantitative analysis results of colony formation assay in HGC27 and AGS cells with indicated treatments of hSULF1 (5 μg/mL, 24h) and SB-431542 (5 μM, 24h). **K and L**. Quantitative analysis results of CDDP IC50 in HGC27 and AGS cells with indicated treatments of hSULF1 (5 μg/mL, 24h) and SB-431542 (5 μM, 24h).
