# Supplementary material for: Cancer associated fibroblasts-derived SULF1 promotes gastric cancer metastasis and CDDP resistance through the TGFBR3-mediated TGF-β signaling pathway
Source: Cell Death Discov. 2024 Mar 4;10:111. doi: 10.1038/s41420-024-01882-y (PMC10912303; doi:10.1038/s41420-024-01882-y)
Supplement: Supplementary file 2 — Supplemental Materials [file 41420_2024_1882_MOESM2_ESM.docx]

**Supplemental Method and Materials**

**Immunofluorescence (IF)**

3×10^5^ cells were seeded in confocal dish (Biosharp, BS-20-GJM) and cultured overnight. After fixation with 4% paraformaldehyde, cells was permeabilized with Triton X-100 (Beyotime, P0096) for 5 min and blocked with Immunol Staining Blocking Buffer (Beyotime, P0102) for 1 h. The cells were incubated with primary antibodies at 4 °C overnight, followed by secondary antibodies incubation for 2 h at room temperature in the dark. Human GC tissue immunofluorescence were preformed using a Multiple Immunofluorescence Kit (AiFang biological, AFIHC035) according to the manufacture’s instruction. Nuclei was stained with DAPI (Beyotime, C1005) and images were captured by LEICA DMi8 system.

Antibodies used for IF were listed in **supplementary Table S4**.

**Immunohistochemical (IHC) staining**

Following the process of antigen retrieval and block, the tissues were subjected to incubation with particular primary antibodies at a temperature of 4 °C for the duration of the overnight period. Subsequently, the tissues were incubated with secondary antibodies for a duration of 1 hour at a temperature of 37 °C. The staining was performed with MaxvisionTM^2^ HRP-Polymer anti-Mouse/Rabbit IHC Kit (MXB Biotechnologies, KIT-5920) according to the instructions. Finally, the sections were stained with hematoxylin (Biosharp, BL702A) and imaged by Nikon microscope. The results were measured using histochemistry score (H-Score, H-SCORE = ∑(pi×i) = percentage of weak intensity×1 + percentage of moderate intensity×2 + percentage of strong intensity×3).

Antibodies used for IHC were listed in **supplementary Table S5**.

**Tunel assay**

The tunel assay was performed with TUNEL BrightRed Apoptosis Detection Kit (Vazyme, A113) according to the manufacturer's instructions. The samples were imaged by LEICA DMi8 system. Results were recorded as percentage of positive cell.

**Transwell assay**

Transwell assay was profermed using 24-well Transwell plates with 8.0 µm pore (Corning Incorporated). 1 × 10^4^ co-cultured GC cells were seeded into upper chambers with none-FBS medium. The lower chamber was supplemented with 600 µL RPMI-1640 medium containing 10% FBS. For the invasive assay, the upper chamber was coated with Matrigel. 48 h later, the migrated or invaded cells were fixed with 4% paraformaldehyde stained with crystal violet.

**Wound-healing assay**

1 × 10^6^ co-cultured GC cells were seeded into 6-well-plate. When cells grew to 90% density, the monolayer was scratched with a sterile 10-μL pipette tip. The monolayer wound images ware captured at 0h and 48h after scratched.

**Cell Counting Kit-8 (CCK-8) assay**

Cells were seeded in 96-well plates at a density of 2000 cells per well, followed by corresponding treatments. CCK-8 reagent (Vazyme, A311) was added for 2h at 37°C, followed by detection at the wavelength of 450 nm with microplate reader (Multiskan FC).

**Colony formation assay**

800 co-cultured GC cells were placed in 6-well plates per well. After incubation at 37°C for 10–14 d, cells were fixed at 4% paraformaldehyde for 10 min and stained with Crystal Violet Staining Solution (Beyotime, C0121) for 30 min.

**Flow cytometric analysis**

For apoptosis assay, co-cultured GC cells were digested by trypsin without EDTA and stained with Annexin V and PI (Vazyme, A211) according to the kit protocols. Then these samples were detected by flow cytometry using the BD FACSCalibur system (BD Biosciences).
