# Supplementary material for: Cancer associated fibroblasts-derived SULF1 promotes gastric cancer metastasis and CDDP resistance through the TGFBR3-mediated TGF-β signaling pathway
Source: Cell Death Discov. 2024 Mar 4;10:111. doi: 10.1038/s41420-024-01882-y (PMC10912303; doi:10.1038/s41420-024-01882-y)
Supplement: Supplementary file 3 — Supplemental Tables [file 41420_2024_1882_MOESM3_ESM.docx]

**Supplemental Tables**

Table S1. Sequences used for RNAi

| Name | Sequence (5^’^ to 3^’^) |
| --- | --- |
| shSULF1#1 | CCCAAATATGAACGGGTCAAA |
| shSULF1#2 | CCAACACATAACTCCTAGTTA |

Table S2. Primers used for qRT-PCR

| Gene | Forward (5^’^ to 3^’^) | Reserves (5^’^ to 3^’^) |
| --- | --- | --- |
| SULF1 | GAGCCATCTTCACCCATTCAA | TTCCCAACCTTATGCCTTGGGT |
| GAPDH | ATCATCAGCAATGCCTCCTG | ATGGACTGTGGTCATGAGTC |

Table S3. Antibodies used for western blotting

| Antibody | Company | Catalog number | Dilution ration |
| --- | --- | --- | --- |
| SULF1 | Invitrogen | PA5-115984 | 1:1000 |
| FAP | abcam | ab207178 | 1:1000 |
| α-SMA | proteintech | 14395-1-AP | 1:5000 |
| N-cad | proteintech | 22018-1-AP | 1:2000 |
| E-cad | proteintech | 20874-1-AP | 1:20000 |
| Vimentin | proteintech | 60330-1-Ig | 1:25000 |
| Slug | proteintech | 12129-1-AP | 1:1000 |
| Snail | proteintech | 13099-1-AP | 1:1000 |
| GAPDH | proteintech | 60004-1-Ig | 1:50000 |
| TGFBR3 | proteintech | 20000-1-AP | 1:1000 |
| TGF‐β1 | proteintech | 21898-1-AP | 1:1000 |
| SMAD2 | proteintech | 12570-1-AP | 1:2000 |
| SMAD3 | proteintech | 66516-1-Ig | 1:2000 |
| p-SMAD2 | Cell Signaling Technology | 18338 | 1:1000 |
| p-SMAD3 | Cell Signaling Technology | 9520 | 1:1000 |

Table S4. Antibodies used for IF

| Antibody | Company | Catalog number | Dilution ration |
| --- | --- | --- | --- |
| SULF1 | Invitrogen | PA5-115984 | 1:200 |
| FAP | abcam | ab207178 | 1:250 |
| α-SMA | proteintech | 14395-1-AP | 1:2000 |
| E-cad | proteintech | 20874-1-AP | 1:500 |
| Vimentin | proteintech | 60330-1-Ig | 1:500 |
| TGFBR3 | proteintech | 20000-1-AP | 1:250 |
| p-SMAD2/3 | abcam | ab254407 | 1:50 |

Table S5. Antibodies used for IHC

| Antibody | Company | Catalog number | Dilution ration |
| --- | --- | --- | --- |
| SULF1 | Invitrogen | PA5-115984 | 1:200 |
| c-caspase3 | Cell Signaling Technology | 9664 | 1:1000 |
| ki67 | abcam | ab92742 | 1:1000 |

Table S6. Antibodies used for IP

| Antibody | Company | Catalog number | Dilution ration |
| --- | --- | --- | --- |
| SULF1 | antibodies | AA 609-871 | 2 μg/mL |
| TGFBR3 | Cell Signaling Technology | 2519 | 2 μg/mL |

Table S7. Reagents used in the experiments

| Reagents | Company | Catalog number |
| --- | --- | --- |
| CDDP | MedChemExpress | HY-17394 |
| TGF-‐β1  SB-431542  hSULF1 | MedChemExpress  MedChemExpress  abcam | HY-P7118  HY-10431  ab153209 |
