# Supplementary figures and images for: Cancer associated fibroblasts-derived SULF1 promotes gastric cancer metastasis and CDDP resistance through the TGFBR3-mediated TGF-β signaling pathway
Source: Cell Death Discov. 2024 Mar 4;10:111. doi: 10.1038/s41420-024-01882-y (PMC10912303; doi:10.1038/s41420-024-01882-y)

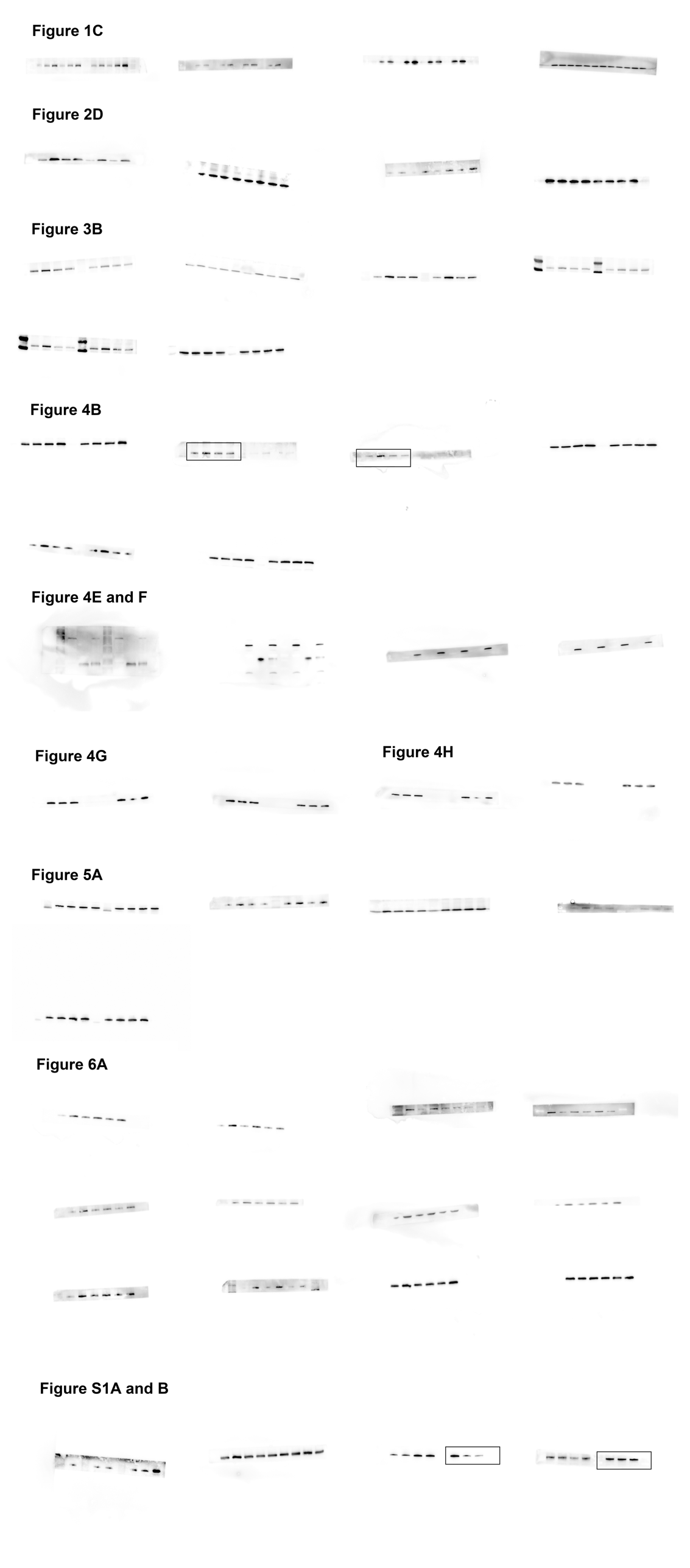

Supplement: Supplementary file 4 — Original Data File [file 41420_2024_1882_MOESM4_ESM.png]
